# London Trauma Conference 2012

**DOI:** 10.1186/1757-7241-21-S1-A1

**Published:** 2013-05-28

**Authors:** HM Lossius, DJ Lockey, GE Davies

**Affiliations:** 1 Norwegian Air Ambulance, Norway; 2 London’s Air Ambulance & Bartshealth NHS Trust, London, UK

## 

The sixth London Trauma Conference was held at the Royal Geographical Society in London between 4^th^ and 7^th^ December. More than 500 delegates and speakers from twenty countries gathered to hear about best practice and innovation in trauma care, pre-hospital care and major incident management. In addition the Norwegian Air Ambulance hosted a day devoted to air ambulance medicine and a parallel symposium examined key aspects of cardiac arrest. This produced to an expanded programme that appealed to a wide range of professional groups. This supplement describes some of the key points made by speakers and also focuses on two innovative aspects of training for penetrating trauma. Firstly resuscitative thoracotomy training for non-surgeons based on a one-day practical workshop held during the conference and secondly the training of young offenders to administer immediate care at the scene of injury – based on an abstract accepted for presentation. The posters and oral presentations submitted cover a wide range of subjects and the accepted abstracts are published at the end of this supplement. Dates for the 2013 conference can be found at http://www.londontraumaconference.com.

